# Histone deacetylase inhibitor, CG200745 attenuates renal fibrosis in obstructive kidney disease

**DOI:** 10.1038/s41598-018-30008-5

**Published:** 2018-08-01

**Authors:** Hong Sang Choi, Ji Hong Song, In Jin Kim, Soo Yeon Joo, Gwang Hyeon Eom, Inkyeom Kim, Hyunju Cha, Joong Myung Cho, Seong Kwon Ma, Soo Wan Kim, Eun Hui Bae

**Affiliations:** 1 Departments of Internal Medicine, Chonnam National University Medical School, Gwangju, 61469 South Korea; 2 Department of Pharmacology, Medical Research Center for Gene Regulation, Chonnam National University Medical School, Gwangju, 61469 South Korea; 3 Department of Pharmacology, School of Medicine, Kyungpook National University, Daegu, 41944 South Korea; 4 CrystalGenomics, Inc., 5 F, Bldg A, Korea Bio Park, Seongnam, 13488 Korea

## Abstract

Tubulointerstitial fibrosis is a common feature of kidney disease. Histone deacetylase (HDAC) inhibitors have been reported to attenuate renal fibrosis progression. Here, we investigated the effect of CG200745, a novel HDAC inhibitor, on renal fibrosis development in a mouse model of unilateral ureteral obstruction (UUO). To examine the effects of CG200745 on renal fibrosis in UUO, C57BL/6 J male mice were divided into three groups: control, UUO, and CG200745 (30 mg/kg/day)-treated UUO groups. CG 200745 was administered through drinking water for 1 week. Human proximal tubular epithelial (HK-2) cells were also treated with CG200745 (10 µM) with or without TGF-β (2 ng/mL). Seven days after UUO, plasma creatinine did not differ among the groups. However, plasma neutrophil gelatinase-associated lipocalin (NGAL) levels were markedly increased in the UUO group, which were attenuated by CG200745 treatment. UUO kidneys developed marked fibrosis as indicated by collagen deposition and increased α-smooth muscle actin (SMA) and fibronectin expression. CG200745 treatment attenuated these fibrotic responses and suppressed UUO-induced production of transforming growth factor-beta1 (TGF-β) and phosphorylation of Smad-2/3. CG200745 treatment also attenuated UUO-induced inflammation as indicated by the expression of inflammatory markers. Furthermore, CG200745 attenuated phosphorylation of p38 mitogen-activated protein kinase in UUO kidneys. In HK-2 cells, TGF-β induced the expression of α-SMA and fibronectin, which were attenuated by CG200745 cotreatment. These results demonstrate that CG200745, a novel HDAC inhibitor, has a renoprotective effect by suppressing renal fibrosis and inflammation in a UUO mouse model.

## Introduction

Although chronic kidney disease (CKD) continues to increase worldwide, treatments are not sufficient to slow the progression of the disease^1^. Neither angiotensin converting enzyme inhibitors (ACEi) nor angiotensin receptor blockers (ARB), which are currently known to delay the progression of CKD, are effective enough. Therefore, studies are underway to find candidate therapeutic agents that can attenuate the progression of CKD, and histone deacetylase (HDAC) inhibitors have been identified as such an agent^2^.

Epigenetic modifications such as DNA methylation or histone acetylation are regarded as important steps in the development of acute kidney injury (AKI), CKD, and the progression of AKI to CKD, and therefore, they have been studied to identify epigenetic changes that occur in kidney injury and therapeutic targets^3^. Histone modifications, which are epigenetic markers that regulate chromatin structure and gene expression, have been studied extensively in relation to kidney damage. In normal cells, histone acetylation is precisely controlled by histone acetyl-transferase (HAT) and HDAC. HDACs are enzymes that remove acetyl groups from histones. HDAC inhibitors mainly act on a zinc domain and cause cell cycle arrest, differentiation, and apoptosis^4^. Therefore, there have been many studies on their use as an anticancer drug, and some HDAC inhibitors have been approved by the Food and Drug Administration (FDA).

Although the main clinical indication of HDAC inhibitors is cancer, they have been shown to have beneficial effects on non-cancerous diseases, including kidney disease. Several HDACs have been reported to be expressed in the developing kidney, renal tubules, and fibroblasts^5–12^. CG200745, (E)-N(1)-(3-(dimethylamino)propyl)-N(8)-hydroxy-2-((naphthalene-1-loxy)methyl)oct-2-enediamide, is a recently developed pan-HDAC inhibitor^13^. CG200745 is known to have a stronger acetylation effect than vorinostat, another pan-HDAC inhibitor. CG200745 not only increases H3 acetylation but also acetylation of non-histone proteins such as tubulin and p53^13,14^. This novel HDAC inhibitor has been shown to have anticancer effects against colon cancer^14^, prostate cancer^13^, non-small cell lung cancer^15^, pancreatic cancer^16^, and cholangiocarcinoma^17^ cell lines. Here, we investigated the renoprotective effects of CG200745 in mouse model of obstructive uropathy.

## Materials and Methods

### Animals

All methods were performed in accordance with the relevant guidelines and regulations. The experimental protocol was approved by the Animal Care Regulations (ACR) Committee of Chonnam National University Medical School (CNU IACUC-H-2017-41). Male 8-week-old C57BL/6 J mice weighing 20~22 g were used for *in vivo* experiments. The mice were divided into three groups: Control (n = 6), unilateral ureteral obstruction (UUO, n = 6), UUO with CG200745 treatment (n = 6). In order to induce the obstructive nephropathy mice model, the operation was performed as follows. After anesthesia induction by using an intraperitoneal injection of ketamine (70 mg/kg), a midline incision was made to expose the abdominal cavity and the left proximal ureter was ligated with 6-0 silk. Control mice were operated in the same way, except that no ligature was made. CG200745 (30 mg/kg/day) was administered to mice via dissolved in water immediately after UUO operation. The mice had free access to standard chow and tap water. Mice were maintained in individual metabolic cages for the last 3 days of the experiment to allow urine collection. After 7 days, the mice were sacrificed, and left kidney was harvested for semiquantitative immunoblotting. Another series of experiment was carried out in the same way for the assay of real-time polymerase chain reaction (PCR) and immunohistochemistry.

### Renal functional parameters

Plasma creatinine was measured using the Jaffe method (Olympus 5431; Olympus Optical, Tokyo, Japan) calibrated for isotope dilution mass spectrometry. Levels of plasma NGAL were determined with a commercial ELISA (R&D Systems, Minneapolis, MN) according to the protocol provided by the manufacturer. A 1:400 sample dilution was performed for plasma NGAL measurement.

### Semiquantitative Immunoblotting

Western blot analysis was performed as previously described^18^. Protein levels were quantified using densitometry. The relative intensities of immunoblot signals were measured by densitometry using Scion image for windows software (Scion Corporation, 2000–2001. version Alpha 4.0.3.2. Maryland, USA) and were expressed as fold changes relative to control.

### Real-Time Polymerase Chain Reaction (Real-Time PCR)

Polymerase chain reaction analysis was performed as previously described^18^. Whole kidney was homogenized in Trizol reagent (Invitrogen, Carlsbad, CA). RNA was extracted with chloroform, precipitated with isopropanol, washed with 75% ethanol, and then dissolved in distilled water. The RNA concentration was determined by the absorbance read at 260 nm (Ultraspec 2000; Pharmacia Biotech, Cambridge, UK). The mRNA expression of inflammatory cytokines and adhesion molecules was determined by real-time PCR. cDNA was made by reverse transcribing 5 μg of total RNA using oligo (dT) priming and superscript reverse transcriptase II (Invitrogen, Carlsbad, CA). cDNA was quantified using Smart Cycler II System (Cepheid, Sunnyvale, CA). SYBR green was used for real-time PCR as dye for detect DNA. Each PCR reaction was done in 10 pM forward primer, 10 pM reverse primer, TOPreal qPCR 2x PreMIX (SYBR Green, Enzynomics, Korea), 1 μg cDNA and H_2_O to bring the final volume to 20 μl. Relative levels of mRNA were determined by real-time PCR, using a Rotor-GeneTM 3000 Detector System (Corbette research, Mortlake, New South Wales, Australia). Sequences of primers were as follows: for GAPDH, 5′-TGTGTCCGTCGTGGATCTGA-3′ (F) and 5′-GATGCCTGCTTCACCACCTT-3′ (R); for α-SMA, 5′-ACTGGGACGACATGGAAAAG-3′ (F) and 5′-CATCTCCAGAGTCCAGCACA-3′ (R); for fibronectin, 5′-ACACGGTTTCCCATTACGCCAT-3′ (F) and 5′-AATGACCACTGCCAAAGCCCAA-3′ (R); for Collagen I, 5′-GAGCGGAGAGTACTGGATCG-3′ (F) and 5′-TACTCGAACGGGAATCCATC-3′ (R); for TGF-β1, 5′-CAACAATTCCTGGCGTTACCTTGG-3′ (F) and 5′-GAAAGCCCTGTATTCCGTCTCCTT-3′ (R); for TNFα, 5′-GCATGATCCGCGACGTGGAA-3′ (F) and 5′-AGATCCATGCCGTTGGCCAG-3′ (R); for MCP-1, 5′-ATCCCAATGAGTAGGCTGGAGAGC-3′ (F) and 5′-CAGAAGTGCTTGAGGTGGTTGTG-3′ (R); for ICAM-1, 5′-AACTTTTCAGCTCCGGTCCTG-3′ (F) and 5′-TCAGTCTGAATTGGACCTGCG-3′ (R); for VCAM-1, 5′-TCTCTCAGGAAATGCCACCC-3′ (F) and 5′-CACAGCCAATAGCAGCACAC-3′ (R).

The PCR was performed according to the following steps: (1) 95 °C for 5 min; (2) 95 °C for 20 s; (3) 58 to 60 °C for 20 s (optimized for each primer pair); (4) 72 °C for 30 s to detect SYBR Green. Steps 2–4 were repeated for additional 40 cycles, while at the end of the last cycle temperature was increased from 60 to 95 °C to produce a melt curve. Data from the reaction were collected and analyzed with the Corbett Research Software. The comparative critical threshold (Ct) values from quadruplicate measurements were used to calculate the gene expression, with normalization to GAPDH as an internal control^19^. Melting curve analysis was performed to enhance specificity of the amplification reaction.

### Histology

Kidney tissues were fixed with 4% paraformaldehyde, embedded in paraffin, and cut into 3 μm-thick sections. Hematoxylin and eosin (H&E) staining was performed to assess the histological morphology. The kidney tissue section slides were incubated in Gill’s hematoxylin for 5 min, washed with tap water, incubated in 95% ethanol, and stained with eosin and phloxine for 1 min. Subsequently, the sections were dehydrated in ethanol and xylene, and were mounted with Canada balsam. For Masson’s trichrome staining, after deparaffinization with xylene, the sections were treated with Bouin’s solution at 56 °C for 30 min and were washed under running tap water until the sections were clear. The sections were subsequently stained with Weigert’s hematoxylin (A:B = 1:1), followed by staining with Biebrich Scarlet/Acid Fuchsin solution for 10 min and washing with distilled water. The sections were incubated with phosphotungstic acid/phosphomolybdic acid solution for 10 min and were treated with Aniline Blue solution for 15 min. They were subsequently incubated with acetic acid for 1 min and were dehydrated with ethanol and xylene. Collagen depositions, nuclei, and muscle fibers were stained blue, black, and red, respectively.

### Cell culture and reagents

Human renal proximal tubular epithelial cells (HK-2 cells, American Type Culture Collection, Manassas, VA, USA) were cultured and passaged every 3~4 days in 100-mm dishes containing combined Dulbecco’s modified Eagle’s (DMEM) and Hams F-12 medium (Welgene, Daegu, Korea) supplemented with 10% fetal bovine serum (FBS; Welgene, Daegu, Korea), 100 U/ml penicillin, and 100 mg/ml streptomycin (Gibco). The cells were then incubated in a humidified atmosphere of 5% CO_2_ and 95% air at 37 °C for 24 h, and sub-cultured until 70–80% confluence. Cells were plated onto 60-mm dishes in a medium containing 10% FBS and incubated for 24 h, following which they were transferred to DMEM-F12 medium with serum free FBS and incubated for an additional 16 h. The cells were then treated with TGF-β (2 ng/ml), either with or without CG200745 (10 μM).

### Chemicals and primary antibodies

CG200745 was generously donated by Crystal Genomics Inc. (Seoul, Rep. Korea). Anti-rabbit antibodies against TGF-β1 (polyclonal; Cell Signaling Technology, MA, USA), extracellular signal regulated kinases 1/2 (ERK 1/2), anti-phosphorylated ERK (p-ERK 1/2), anti-c-Jun N-terminal kinase (JNK), anti-phosphorylated JNK (p-JNK), anti-total p38, anti-phosphorylated p38 (p-p38), Smad2/3, and phosphorylated Smad2/3 (Cell Signaling Technology, MA, USA), anti-mouse antibodies against GAPDH (clone GAPDH-71.1;monoclonal), α-SMA (1A4 Clone; monoclonal; Sigma Chemical Co. St. Louis, MO, USA), fibronectin (BD Biosciences, San Jose, CA, USA), heme oxygenase-1 (HO-1, Abcam, Inc., Cambridge, MA, USA), and F4/80 (AbD Serotec, Raleigh, NC, USA) were commercially obtained.

### Statistical Analysis

The results were expressed as mean ± standard error of the mean (SEM). Multiple comparisons among the 3 groups were performed using one-way analysis of variance (ANOVA) and the *post-hoc* Tukey’s honestly significant difference test. Differences with values of p < 0.05 were considered significant.

## Results

### Functional parameters

Table 1 shows the changes in the functional parameters. UUO caused an increase in urine volume and kidney weight to body weight ratio, but CG200745 treatment did not significantly alleviate this change. Notably, plasma creatinine was higher in the UUO model than in the control, and the increase in plasma NGAL in UUO mice was attenuated after co-treatment with CG200745.

**Table 1:** Effect of CG200745 on real function.

	Control	UUO	UUO + CG
BW (g)	23.9 ± 0.12	23.9 ± 0.64	24.5 ± 0.53
KW/BW (g/kg)	5.7 ± 0.18	6.6 ± 0.23^*^	6.0 ± 0.16
UO (ml/day)	2.2 ± 0.15	3.4 ± 0.28^*^	4.6 ± 0.89
BUN (mg/dl)	28.2 ± 0.40	26.6 ± 1.71	30.1 ± 2.08
P_Cr_ (mg/dl)	0.09 ± 0.01	0.152 ± 0.03	0.103 ± 0.02
Plasma NGAL (ng/ml)	461.0 ± 27.8	2155.7 ± 387.7^*^	701.0 ± 153.0^#^

Abbreviations: UUO, unilateral ureteral obstruction; CG, CG200745; BW, body weight; KW, kidney weight; UO, urine output; BUN, blood urea nitrogen; P_Cr_, plasma creatinine; NGAL, neutrophil gelatinase-associated lipocalin.

^*^p < 0.05 compared with control. ^#^p < 0.05 compared with UUO. Values are expressed as the mean ± SEM.

### CG200745 attenuated morphological changes in UUO kidneys

**Figure 1:**
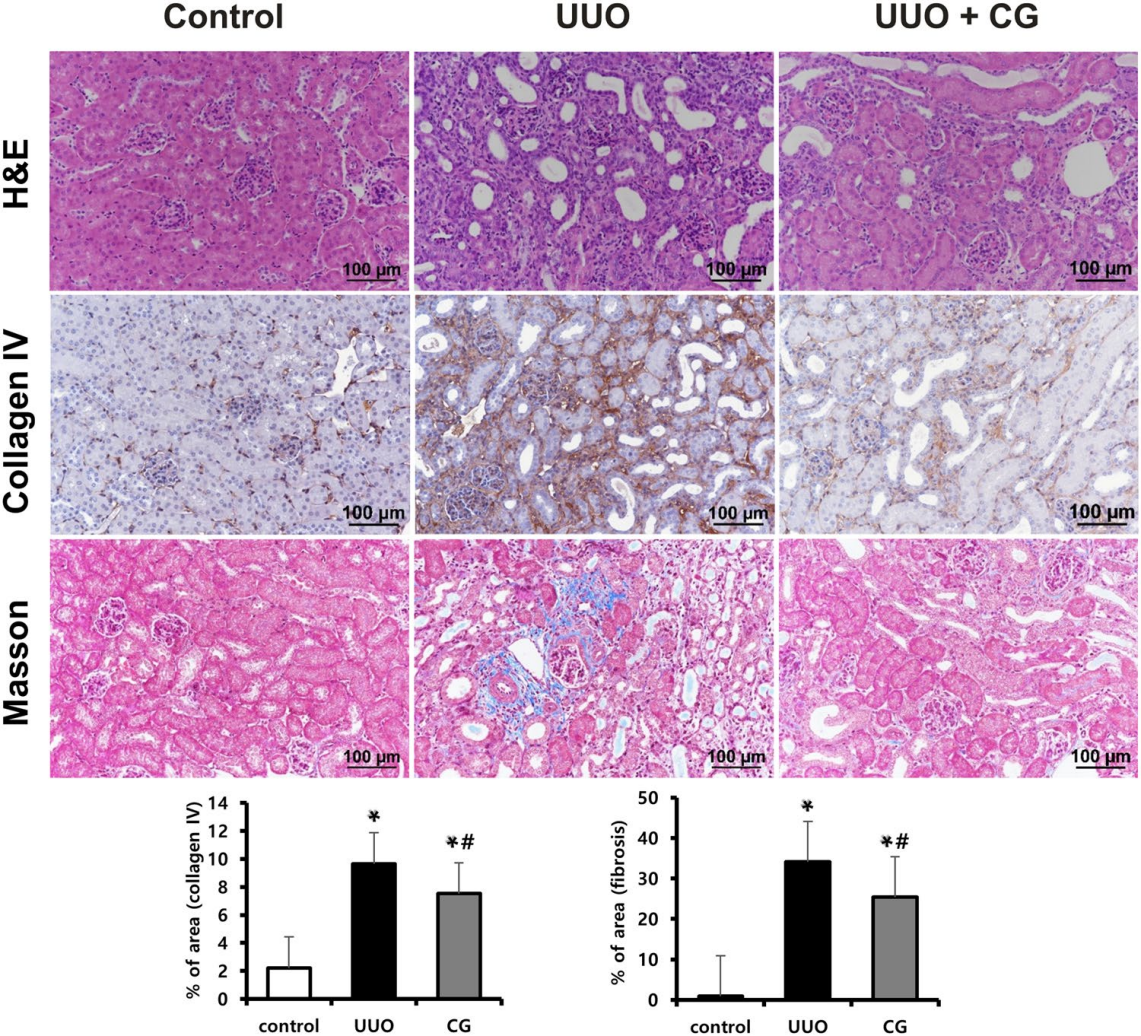
Effects of CG200745 on morphological changes in obstructed mouse kidneys. C57BL/6 mice were administered CG200745 (30 mg/kg/day) via drinking water, immediately after unilateral ureteral obstruction (UUO) operation. The UUO lasted for 7 days. Hematoxylin and eosin (H&E) staining revealed the presence of tubular casts and obstructions in the kidney of UUO mice, which was attenuated by CG200745 treatment (first row). Representative immunohistochemical staining using anti-collagen type IV antibody (second row) and Masson’s trichrome staining was performed to examine interstitial fibrosis (third row). Scale bar is 100 μm.

Severe morphological changes were observed in UUO kidneys compared to control kidneys (Fig. 1). Hematoxylin and eosin staining revealed tubular dilatation, atrophy, interstitial infiltration of mononuclear cells, and interstitial fibrosis in obstructed kidneys relative to control kidneys. However, these changes were attenuated by CG200745 treatment.

### CG200745 ameliorated kidney fibrosis in UUO kidneys

**Figure 2:**
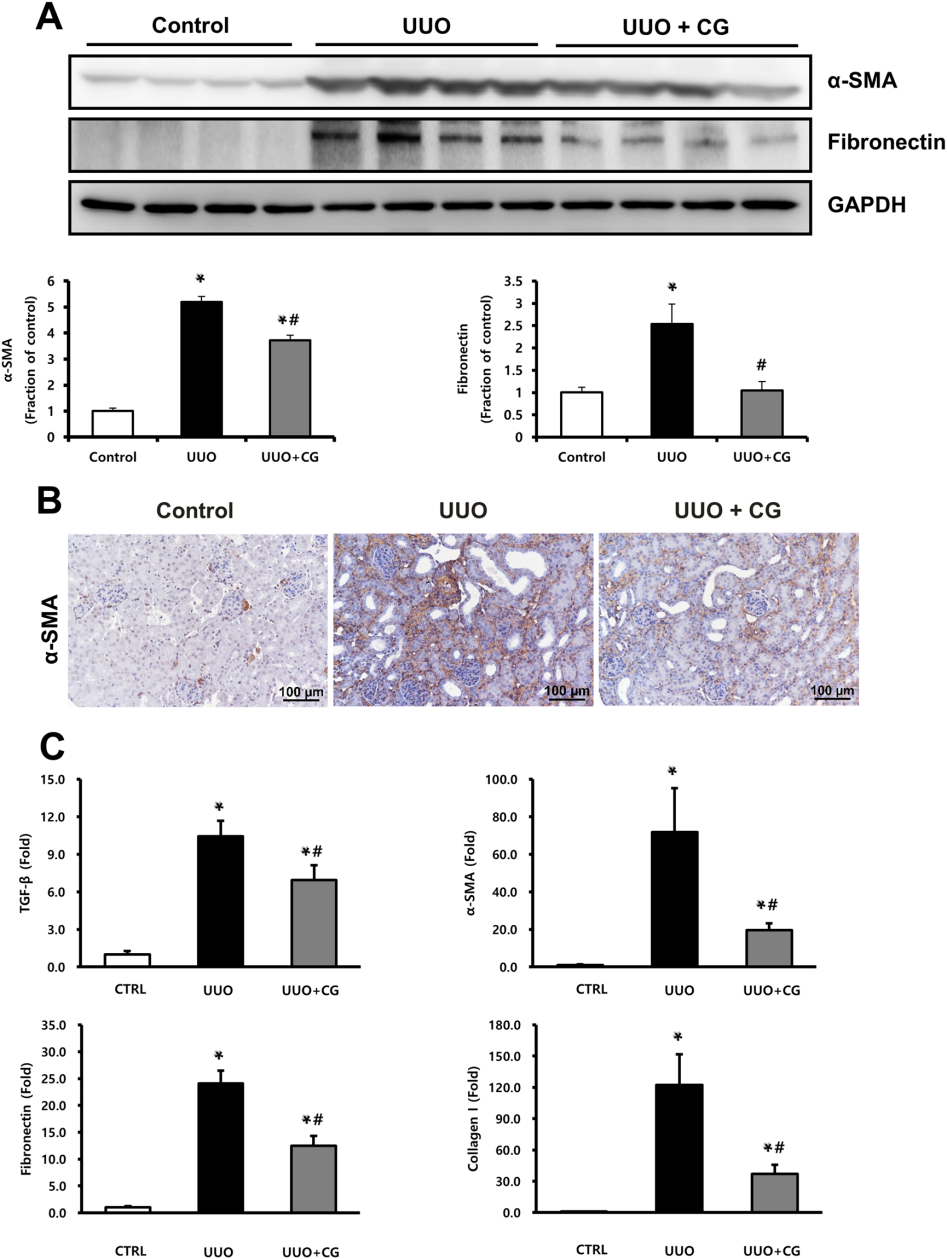
Effects of CG200745 on epithelial-mesenchymal transition and fibrosis in obstructed mouse kidney. (**A**) Protein expression of the α-SMA and fibronectin was assessed in UUO kidneys and unaffected controls. Each column represents mean ± SEM. *p < 0.05, compared with the control. ^#^p < 0.05, compared with the UUO group. (**B**) Representative immunohistochemical staining of α-SMA in UUO mice. Scale bar is 100 μm. (**C**) mRNA expression of markers of fibrosis, TGF-β, α-SMA, fibronectin and collagen I. Each column represents mean ± SEM. *p < 0.05, compared with the control. ^#^p < 0.05, compared with the UUO group.

We performed Masson’s trichrome staining to determine whether CG200745 can function a therapeutic agent for renal fibrosis. As shown in Fig. 1, deposition of interstitial collagen was observed in UUO kidneys and was attenuated by CG200745 treatment. Immunohistochemical staining for type IV collagen revealed the increased accumulation of type IV collagen in the peritubular and periglomerular interstitium in UUO kidneys, which was attenuated by CG200745 treatment (Fig. 1). We investigated the effects of CG200745 on the expression of the myofibroblast molecular marker α-SMA and fibronectin. In obstructed kidneys, the expression of α-SMA and fibronectin increased, which was prevented by CG200745 treatment (Fig. 2A). Immunohistochemical staining for α-SMA revealed its increased expression in the peritubular and periglomerular interstitium in UUO kidneys, which was significantly reduced by CG200745 treatment (Fig. 2B). We also investigated the mRNA expression of α-SMA, fibronectin, collagen I, and TGF-β. UUO significantly increased renal α-SMA, fibronectin, collagen I, and TGF-β mRNA expression, and these changes were attenuated by CG200745 co-treatment (Fig. 2C).

### CG200745 inhibited the TGF-β/Smad pathway and p38-MAPK activation in UUO kidneys

**Figure 3:**
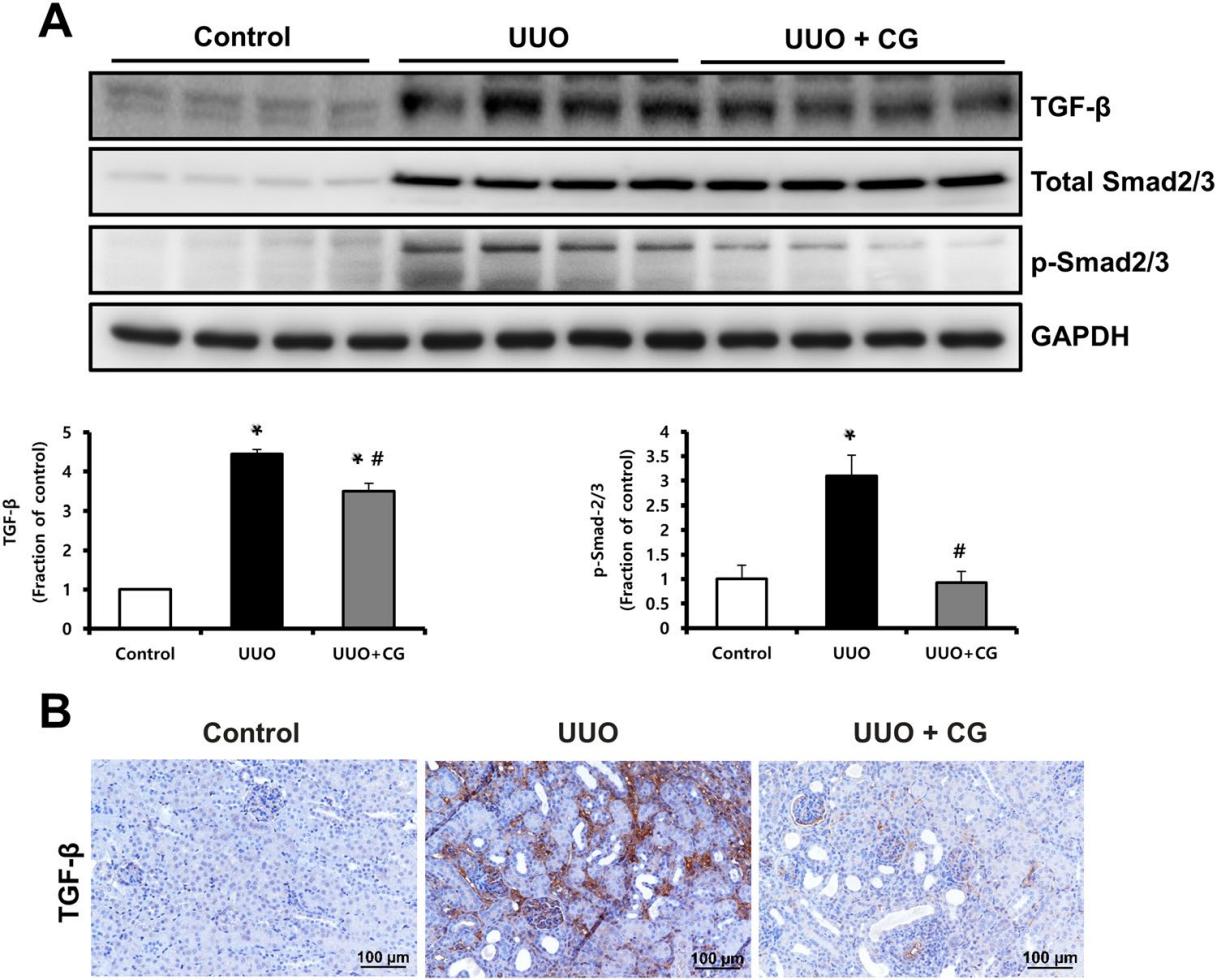
Effects of CG200745 on TGF-β/Smad pathway in obstructed mouse kidney. (**A**) Protein expression of the TGF-β and Smad2/3 was assessed in UUO kidneys and unaffected controls. Each column represents mean ± SEM. *p < 0.05, compared with the control. ^#^p < 0.05, compared with the UUO group. (**B**) Representative immunohistochemical staining of TGF-β in UUO mice. Scale bar is 100 μm.

**Figure 4:**
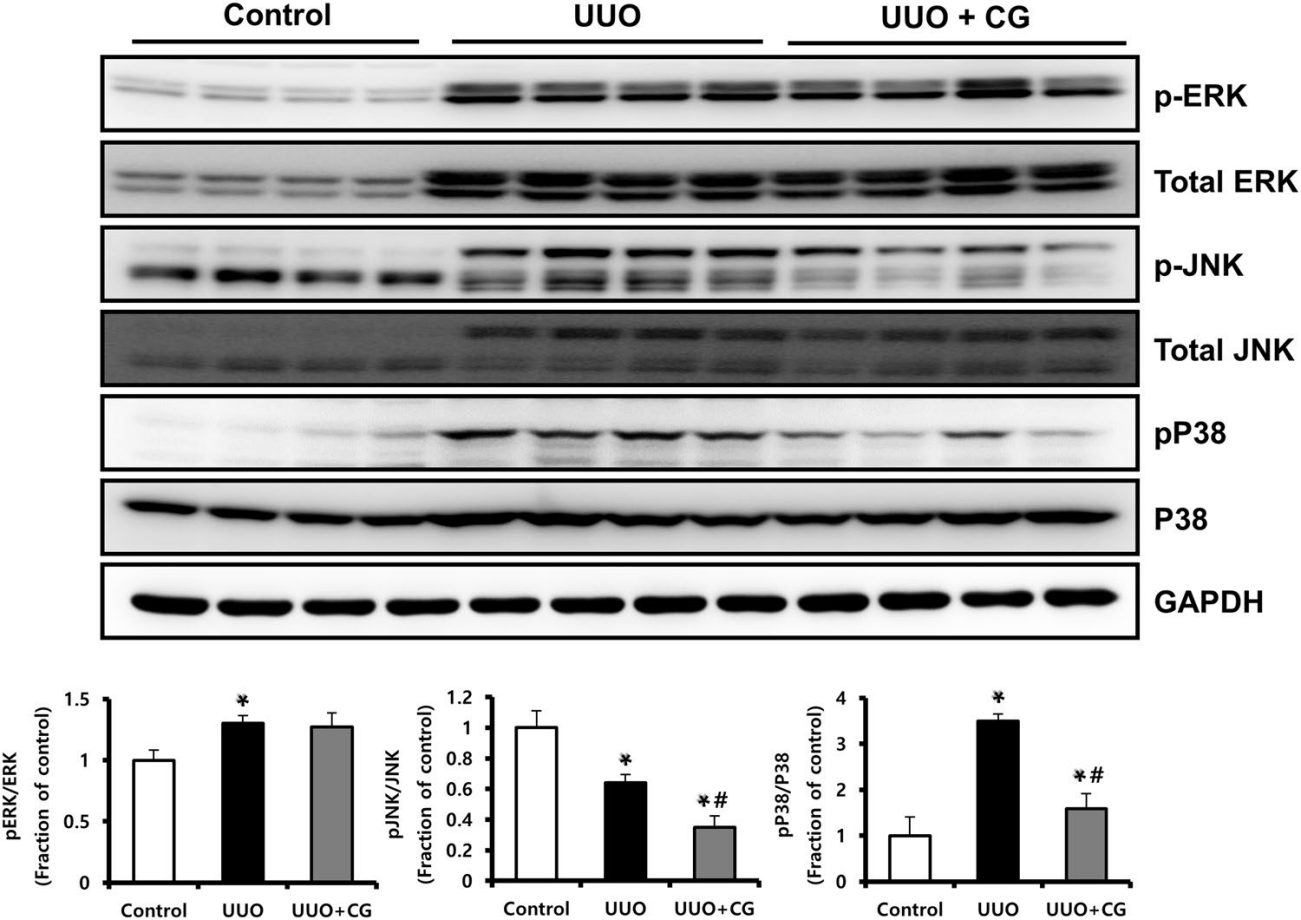
Effects of CG200745 on p38 MAPK pathway in obstructed mouse kidney. Protein expression of the total ERK, phosphorylated ERK (p-ERK), total JNK, phosphorylated JNK (p-JNK), P38 and phosphorylated P38 (pP38) was assessed in UUO kidneys and unaffected controls. Each column represents ratio of pP38 to P38 (pP38/P38) as mean ± SEM. *p < 0.05, compared with the control. ^#^p < 0.05, compared with the UUO group.

We performed immunohistochemical staining and western blot analysis to determine whether CG200745 affected TGF-β/Smad signaling, which is a critical mediator of renal fibrosis. Immunohistochemical staining for TGF-β revealed the increased expression of TGF-β in UUO kidneys, which was attenuated by CG200745 treatment (Fig. 3B). In the western blot analysis, protein expression of TGF-β and phosphorylated Smad2/3 significantly increased in UUO kidneys (Fig. 3A), which were attenuated by CG200745 treatment. TGF-β/Smad signaling interacts with MAPK signaling in renal fibrosis^20–23^. To determine whether CG200745 affects TGF-β-induced MAPK signaling, we assessed the protein expression of JNK, ERK, and p38 by western blotting in UUO kidneys. The phosphorylated forms of JNK, ERK, and p38 increased in UUO kidneys compared to those in control kidneys (Fig. 4). The expression of phosphorylated JNK and p38 was reduced by CG200745 treatment, but not phosphorylated ERK. We observed that the expression of non-phosphorylated ERK and JNK also increased in UUO kidneys compared to that in control kidneys. Finally, the ratio of phosphorylated p38 to total p38 significantly decreased in CG200745-treated UUO kidneys compared to that in control kidneys.

### CG200745 attenuates oxidative stress, inflammatory cytokines, and adhesion molecules in UUO kidneys

**Figure 5:**
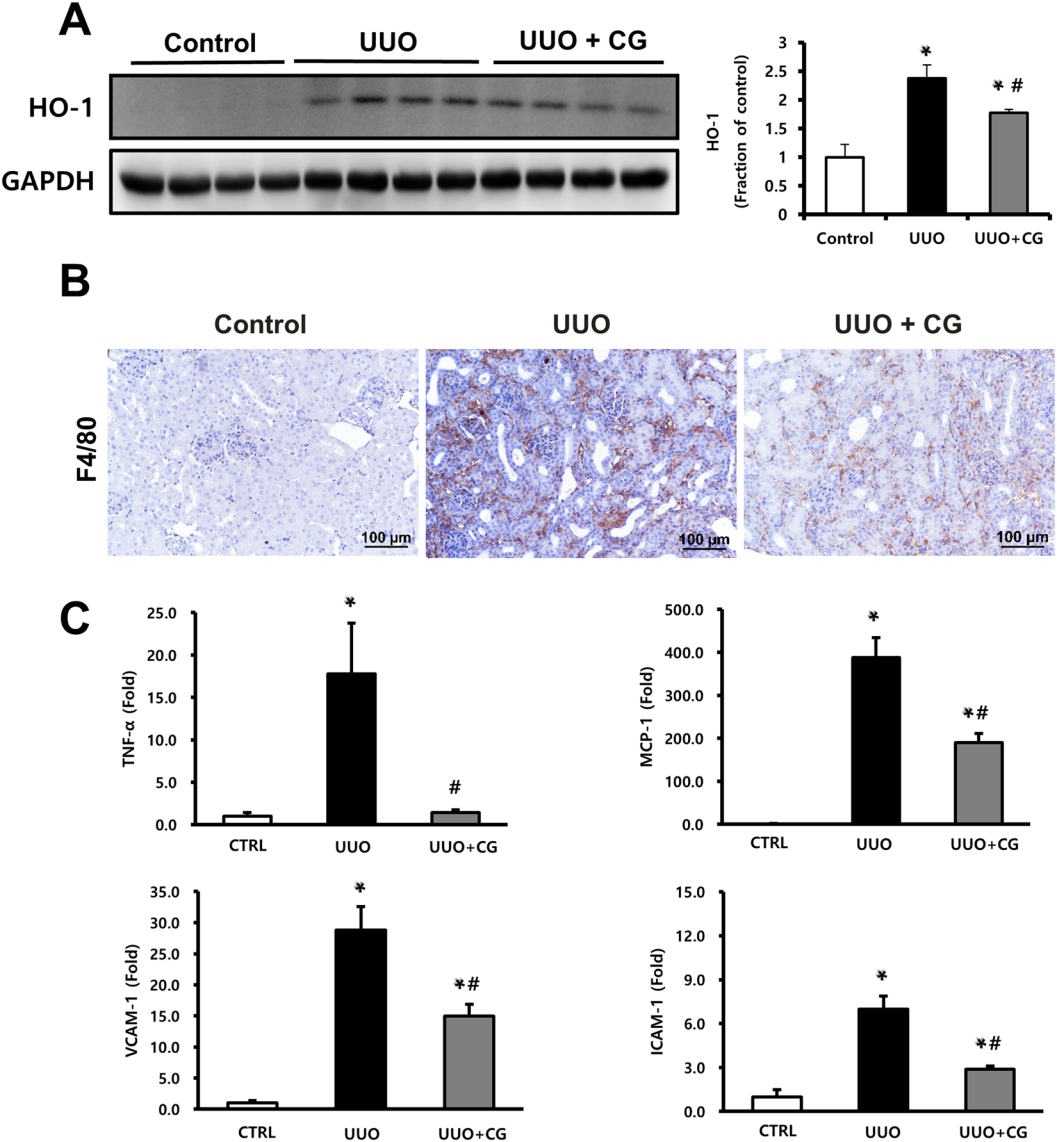
Effects of CG200745 on oxidative stress, inflammatory cytokines and adhesion molecules in obstructed mouse kidney. (**A**) Protein expression of the heme oxygenase 1 (HO-1) was assessed in UUO kidneys and unaffected controls. Each column represents mean ± SEM. *p < 0.05, compared with the control. ^#^p < 0.05, compared with the UUO group. (**B**) Representative immunohistochemical staining of F4/80 in UUO mice. Scale bar is 100 μm. (**C**) mRNA expression of markers of inflammation, TNF-α, MCP-1, VCAM-1 and ICAM-1. Each column represents mean ± SEM. *p < 0.05, compared with the control. ^#^p < 0.05, compared with the UUO group.

To evaluate UUO-induced oxidative stress, we measured HO-1 levels in kidney tissues. We found increased expression of HO-1 in UUO kidneys, which was attenuated by CG200745 treatment (Fig. 5A). Immunohistochemical staining for F4/80, a marker of murine macrophage populations, revealed its increased expression in UUO kidneys, which was attenuated by CG200745 treatment (Fig. 5B). We also investigated the expression of TNF-α, a key inflammatory cytokine produced by infiltrating cells. UUO significantly induced renal TNF-α mRNA expression, but these changes were attenuated by CG200745 treatment (Fig. 5C). Increased expression of certain chemokines and adhesion molecules such as MCP-1, ICAM-1, and VCAM-1 that can activate, recruit, or induce the transmigration of inflammatory cells to the site of kidney injury was also seen. The expression of these factors was induced by UUO, and CG200745 treatment significantly reduced their expression in UUO kidneys (Fig. 5C).

### CG200745 attenuates markers of fibrosis in TGF-β induced HK2 cell injury

**Figure 6:**
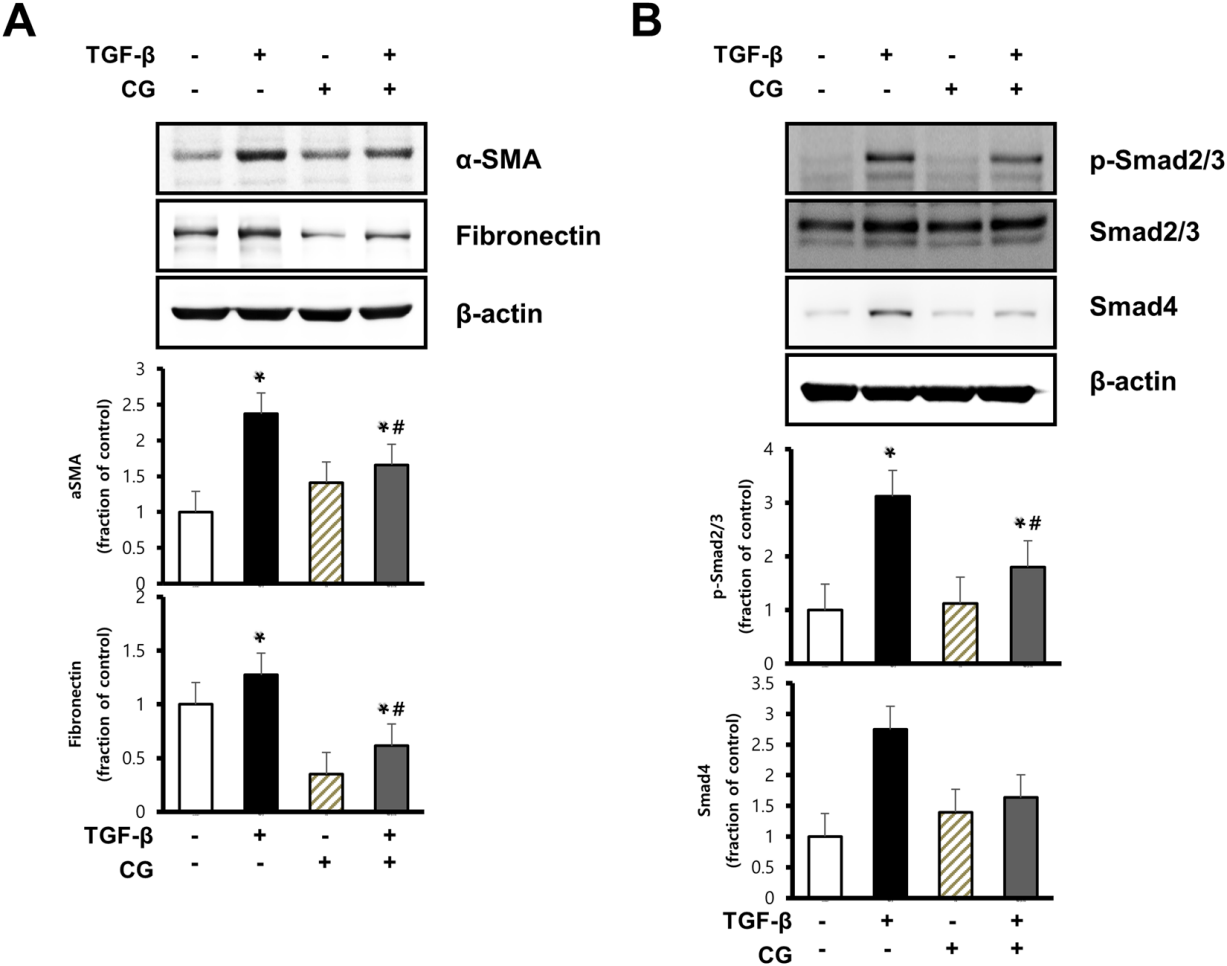
Effects of CG200745 on epithelial-mesenchymal transition and fibrosis in TGF-β treated HK-2 cell. HK-2 cells were exposed to TGF-β (2 ng/ml, 16 h) with or without treatment with CG200745 (10 μM). (**A**) Protein expression of the α-SMA and fibronectin were analyzed. (**B**) Protein expression of the Smad2/3 and Smad4 were analyzed. Each column represents mean ± SEM. *p < 0.05, compared with the control. ^#^p < 0.05, compared with TGF-β treated HK-2 cells.

*In vitro* studies showed that CG200745 treatment counteracted the TGF-β-induced upregulation of α-SMA and fibronectin (Fig. 6A). The expression of phosphorylated Smad 2/3, a downstream signal of TGF-β, increased in TGF-β-treated cells and decreased after CG200745 co-treatment (Fig. 6B).

## Discussion

Here, we demonstrated that CG200745 attenuates renal injury in a mouse model of UUO. As mentioned above, CG200745 reduced renal fibrosis caused by UUO. In addition, CG200745 also inhibited the TGF-β/Smad-dependent signaling pathway that contributes to renal fibrosis induced by UUO. CG200745 treatment also reduced oxidative stress and inflammatory cytokines that contribute to renal damage caused by UUO. These findings suggest that CG200745 prevents UUO-induced kidney injury and may be useful as a therapeutic agent to treat kidney disease.

The UUO model is a representative animal model of obstructive nephropathy that is characterized by progressive tubular-interstitial fibrosis^24^. In this model, the tubular-interstitial inflammation and fibrosis that are observed in human obstructive nephropathy are reproduced in a very similar manner. Extracellular matrix accumulation in the interstitial space and tubular atrophy are common histopathological features of progressive renal disease due to various causes. Therefore, UUO is a very suitable model for studying the pathophysiology of chronic kidney disease and for evaluating the potential of therapeutic candidates.

HDAC abnormalities are known to be associated with many diseases, including kidney disease^25^. Initially, HDAC inhibitors were mostly studied for their anticancer effects. However, HDAC inhibitors were shown to have beneficial effects on renal diseases, with antiinflammatory and anti-fibrosis effects^2^. Pharmacological inhibition of HDAC has been reported to attenuate the progression of renal fibrogenesis in obstructed kidneys^4,6,26–28^ and to reduce cyst formation in polycystic kidney disease^10,29,30^. HDAC inhibitors are also able to ameliorate renal lesions in diabetes nephropathy^31–34^, lupus nephritis^35,36^, aristolochic acid nephropathy^37^, and transplant nephropathy^38^. In particular, HDAC inhibition in the UUO model has been shown to inhibit renal fibrosis and inflammation and to reduce apoptosis in tubular cells^27,28^. It has been reported that this inhibition of renal fibrosis occurs by regulating a pathway through TGF-β^26^. Therefore, we hypothesized that a newly developed HDAC inhibitor, CG200745, would have a renoprotective effect and be a potential drug to inhibit the progression of chronic kidney disease.

In UUO kidneys, severe tubulointerstitial fibrosis and extracellular matrix accumulation were observed. However, with CG200745 treatment, renal injury and fibrosis were significantly attenuated. Histologic findings showed that CG200745 treatment not only partially reduced the morphologically changes in UUO kidneys but also markedly reduced ECM accumulation (Fig. 1). UUO led to the marked up-regulation of α-SMA, a marker of activated myofibroblasts and fibronectin, a glycoprotein in the extracellular matrix, whereas CG200745 treatment ameliorated these effects. CG200745 decreased the mRNA expression of α-SMA, fibronectin, and collagen I in the UUO kidney, indicating that CG200745 suppressed renal fibrosis at the gene expression level.

TGF-β is a key mediator in renal fibrosis^39^, and TGF-β/Smad signaling is a major intracellular signaling pathway of TGF-β action in progressive renal fibrosis. When TGF-β binds to the type II TGF-β receptor, it recruits type I TGF-β receptors and phosphorylates Smad2 and Smad3^40^. The phosphorylated Smad2/3 complex translocates into the nucleus to regulate the transcription of target genes related to fibrosis. Here, we found that TGF-β expression in the interstitium of UUO kidneys significantly increased and then decreased after treatment with CG200745, and the same results were obtained with western blotting. Phosphorylated Smad2/3 also showed the same trend, suggesting that Smad signaling is involved in the mechanism of the attenuation of kidney fibrosis by CG200745. Additionally, TGF-β can function through Smad-independent pathways such as the MAPK pathway^41^. Activated receptor complex of TGF-β is known to activate three parallel signal transduction MAPK pathways, involving ERK, JNK, and P38^42^. We found that phosphorylated P38 increased in UUO kidneys but improved after CG200745 treatment. Therefore, CG200745 regulates TGF-β signaling via p38 MAPK.

HO-1 is an enzyme that degrades heme and is responsible for protecting tissues from damage induced by oxidative insults. HO-1 deficiency has been associated with increased fibrosis, tubular TGF-β expression, and inflammation in obstructive kidney disease^43^. In our study, we found an increase in HO-1 as in previous studies but found a decrease in expression after treatment with CG200745. Macrophages play a role in the secretion of cytokines associated with renal fibrosis. As shown in Fig. 5B, in the UUO kidney, the number of cells positive for F4/80, a marker of the murine macrophage population, significantly increased, but it significantly decreased after treatment with CG200745. In general, enhanced local expression of proinflammatory cytokines, chemokine receptors, and adhesion molecules is preceded by macrophage infiltration^44^. In our study, the expression of TNF-α, MCP-1, VCAM-1, and ICAM-1 increased in UUO kidneys but was ameliorated by CG200745 treatment. Therefore, CG200745 can prevent renal damage through antioxidative and anti-inflammatory effects.

In summary, the novel HDAC inhibitor CG200745 had a kidney protective effect by reducing renal fibrosis and inflammation in a model of obstructive nephropathy. CG200745 thus has potential as a novel therapeutic agent for inhibiting the progression of chronic kidney disease.
